# Italian university students’ future time perspective and well-being during the COVID-19 pandemic

**DOI:** 10.3389/fpsyg.2024.1404952

**Published:** 2024-07-23

**Authors:** Santa Parrello, Jacopo Postiglione, Luigia Simona Sica, Barbara De Rosa, Anna Parola, Giorgio Maria Regnoli, Elisabetta Fenizia, Massimiliano Sommantico

**Affiliations:** Department of Humanities, Section of Psychology and Educational Sciences, University of Naples “Federico II”, Naples, Italy

**Keywords:** future time perspective, COVID-19 pandemic, young adults, university students, optimism, well-being

## Abstract

**Introduction:**

During the COVID-19 pandemic, young adults worldwide showed signs of distress as they were affected in their specific developmental tasks, including the construction of personal and professional futures.

**Methods:**

The present study aimed to assess the situational future time perspective of Italian university students during the second pandemic wave, as measured by an *ad hoc* constructed instrument, to explore its interaction with some dispositional traits relevant in future construction, such as optimism, sense of life, aggression, and dispositional future time perspective, and to test their effect on psychological well-being. The total sample consisted of 389 subjects (18–35 years, *M* = 23.5, *SD* = 4.4).

**Results and discussion:**

The results indicated that the pandemic experience, assessed by surveying specific indicators, negatively affected the future time perspective of students, particularly those dispositionally optimistic and convinced that life has meaning. However, awareness of the negative impact that the pandemic brought to the vision of the future seems to have dampened the levels of depression and stress, while anxiety was found to be related only to dispositional traits. The results also suggested the need for educational and economic policies that help young adults develop confidence in the future and in their ability to build it.

## 1 Introduction

Pandemics are sudden collective events that psychological research associates with uncertainty, fear for one’s safety and economic security, loss of control over one’s life, and emotional distress. They thus take the form of serious environmental risk factors for people’s psychological well-being and mental health (Tucci et al., 2017). However, each individual can also leverage specific protective factors, as well as individual, relational, and cultural resources that can support him or her in coping with a potentially traumatic situation by cushioning its impact (Pfefferbaum et al., 2007; Rimé, 2020).

The COVID-19 pandemic, which broke out in 2020, showed all the characteristics of a traumatic event of a collective nature, capable of negatively affecting psychological well-being and mental health. Indeed, increased levels of anxiety, depression, posttraumatic symptoms, compulsive behaviors, and social isolation have been reported in individuals of all ages in countries around the world (e.g., Brooks et al., 2020; Horesh and Brown, 2020; Parola et al., 2020; De Rosa, 2021; Kira et al., 2021; Sommantico et al., 2021; De Rosa and Regnoli, 2022; Lacatena and Sommantico, 2022; Regnoli et al., 2022, 2023; Rossi et al., 2023).

Adolescents and young adults showed signs of reduced psychological well-being and an overall deterioration in quality of life due to the accumulation of pandemic risk factors related to their age-specific developmental tasks, such as the need for autonomy from parents, social experimentation, and identity construction (e.g., Cao et al., 2020; Charles et al., 2021; Magson et al., 2021; Varma et al., 2021; Giotsa, 2022; Ludwig-Walz et al., 2022; Zhen and Zhou, 2022; Zurlo et al., 2022). In academic contexts, they had to face rapid changes in their learning experiences, due to the distance learning imposed by lockdown restrictions. In particular, several studies have explored the impact of pandemic on university students’ mental health taking account that higher education students well-being has been a growing research field in the last years (e.g., Chen and Lucock, 2022; Gómez-García et al., 2022).

In general, the home confinement imposed to limit contagion has affected their need for extrafamiliar social relationships (e.g., Chen et al., 2020; Lardone et al., 2020; Parola et al., 2020; Sahu, 2020; Parrello et al., 2021; Boursier et al., 2023), prompting them to make greater use of digital devices, as well as social media for maintaining, albeit virtual, interpersonal relationships with peers (Galvin et al., 2022; Schauffel et al., 2022), and forcing them to be in familiar environments often charged with emotional tension and conflict (Usher et al., 2020). Furthermore, university students missed the opportunity to celebrate their graduation ceremonies, as well as to leave for their study exchange programs. In addition, they had to renounce to work experiences and to deal with an increased unpredictability regarding their career expectations (Chen and Lucock, 2022).

Forced confinement drastically altered daily routines, significantly affecting their relationship with time (Holman and Grisham, 2020; Micillo et al., 2022). Indeed, time was often perceived as too slow, too fast, or confusing, no longer being marked by the usual daily rhythms and commitments, such as sleep–wake, school, work, sports, and outings (e.g., Wittmann, 2020; Shoham, 2021; Sommantico et al., 2021), causing them to experience disorientation, boredom (e.g., Cellini et al., 2020; Droit-Volet et al., 2020; Ogden, 2020; Cravo et al., 2022), and anxiety about loss of control (Nielsen et al., 2021). Furthermore, the media narrative has made it difficult to distract from the theme of a future characterized by severe health, social, and economic uncertainty, consistently showing a horizon of risk of economic recession, with increased competition for jobs (Ranta et al., 2020; Montenovo et al., 2022; Nuckols et al., 2023), as well as the risk of illness and death (Garfin et al., 2020; Magson et al., 2021; Rupprecht et al., 2022). Moreover, many students, after completing secondary school, felt that their lives were as if “on hold or “stuck,” while for others—especially those from disadvantaged socioeconomic backgrounds—the need to survive the pandemic economically became a more important factor in career decisions than personal interests, dreams, or ambitions (Carey et al., 2023).

In the context of the COVID-19 pandemic, psychological resources, such as positive perception of the future, finding meaning in life, and optimism may be extremely important for coping with the difficult and new situation and for maintaining psychological well-being. Among them, positive psychology indicates Time Perspective (TP) as a central aspect of human daily psychological functioning. Namely, positive future orientation is strongly associated with a range of various mental well-being indicators.

According to this view, future time perspective—understood as the set of thoughts, fantasies, and feelings that individuals direct toward their future—has been investigated from several (Kooij et al., 2018), not mutually exclusive, theoretical models: (a) the Three-Process Model (Nurmi, 1989), according to which the temporal perspective has a cognitive, motivational/affective, and behavioral component; (b) the Time Perspective Model (Zimbardo and Boyd, 1999), focusing on the individual’s perception of past, present, and future; and (c) the Possible Selves Model (Markus and Nurius, 1986), according to which the future perspective contains different representations of self, such as the feared Self, the expected Self, and the hoped-for Self. Regardless of the underlying theoretical model, studies on the subject show that the future time perspective is the basis for identifying goals and making plans to achieve them and is associated with factors of well-being in several domains, such as emotional regulation, frustration tolerance, school and vocational performance, and health promotion (e.g., Nuttin, 1985; Dittmann-Kohli, 1986; Zimbardo and Boyd, 1999; McInerney, 2004; Shipp et al., 2009; Stolarski et al., 2015, 2020; Villani et al., 2020). For this reason, it is considered an important developmental task of the late adolescent and young adult grappling with the process of identity construction (Crocetti et al., 2013). It is also considered a dispositional stable trait of the individual, formed throughout life, based on the developmental context, and capable of influencing reactions to the environment (Lyu and Huang, 2016; Zaleski et al., 2019). Indeed, young people who lack positive expectations of their future are also those most prone to high-risk situations (Baños et al., 2017).

The COVID-19 pandemic appears to have significantly affected the future time perspective of adolescents and young people, amplifying feelings of uncertainty, anxiety, and worry about the safety of themselves and their families, the world economy, and the future of democracy, as well as the imminence of disasters and the role such disasters may play in hindering the achievement of their individual goals (Carey et al., 2023).

In this regard, for example, Loose and Vásquez-Echeverría (2021) and Loose et al. (2022) questioned how dispositional temporal perspectives might have affected college students’ ability to cope with the COVID-19 pandemic while preserving their well-being, but also whether the pandemic experience was powerful enough to change their temporal perspective. Indeed, it has already happened that future orientation—understood as a positive future time perspective—decreased in the population after the 9/11 terrorist attacks (Holman and Silver, 2005; Holman et al., 2016), as well as in Israeli and Palestinian adolescents exposed to war events of traumatic magnitude (Seginer and Schlesinger, 1998; Solomon and Lavi, 2005). In the study by Loose et al. (2022), the pandemic did not appear to have had similar effects, perhaps because, as the authors comment, it was carried out at a stage that was not particularly dramatic. Indeed, 60% of the students involved reported thinking more about the future since the beginning of COVID-19, 40% about the present, and 22% about the past, showing more psychological distress and learning difficulties in the latter case.

Thus, if a positive future time perspective seems to be a significant protective factor for students, preserving planning and hopes even under difficult conditions such as a pandemic (e.g., Commodari and La Rosa, 2020; Fioretti et al., 2020; Ding and Li, 2023), other studies have shown that during a pandemic a “here and now”-oriented temporal perspective, when accompanied by the belief that the present is rich in value and that current behaviors are determinative for the future, helped adhere to public health protection rules (Sobol et al., 2020). Furthermore, this same temporal perspective helps to maintain better subjective well-being when associated with certain personality traits, such as extroversion and neuroticism (Mioni et al., 2022). Further research has shown, however, that a negative future time perspective can also play a protective function when associated with the perception of high risk and one’s vulnerability to COVID-19, prompting one to adopt preventive behaviors (Zancu et al., 2022).

Cultural contexts are also relevant. Indeed, results from the cross-cultural study by Micillo et al. (2022) on time perspective of individuals aged 18–60 years during the pandemic in 6 different countries, show that younger participants reported lower scores on the future time perspective subscale than older individuals, as did Italians compared to French and Argentines. Furthermore, future orientation was a significant negative predictor for depression in Japan, while it positively predicted anxiety in Italy and Turkey. This could be explained in light of some socioeconomic and cultural characteristics specific to the Italian context, such as children’s long cohabitation with their parents, and prolonged education, But it is also important consider Italian social policies that do not provide great resources to families and young people, as well as high levels of unemployment, delayed entry into the labor market, and media portrayal of a catastrophic future (D’Agostino and Regoli, 2013; Parrello, 2018).

Italy, moreover, was the first country after China to be suddenly and severely affected by the COVID-19 pandemic, with a very high number of sick and dead. Indeed, as early as March 2020, the government imposed a strict lockdown on the entire population, affecting public economic, cultural, and educational activities, which were replaced by remote work and distance education wherever possible. Individuals were forbidden to leave their homes except in cases of extreme necessity, thus imposing a drastic form of isolation and a modification of social routines unprecedented in history. The lockdown also led to the loss of many jobs and a rapid increase in poverty rates. After a brief interruption, during the second pandemic wave in the spring of 2021, the restrictive measures were reinstated only in geographic areas at greatest risk of contagion, referred to as “red zones.” Adolescents and young adults were the most affected by the restrictions imposed and were also “blamed” in the media narrative that considered them the main potential carriers of contagion in families (Cornaggia et al., 2023). In this sense, the data on their well-being and mental health immediately appeared worrisome, the phenomena of dropping out of educational paths having increased significantly (e.g., Carey et al., 2023; Delgado et al., 2023; Parola et al., 2023; Roque Pimentel et al., 2023) Furthermore, signs of malaise such as anxiety, depression, withdrawal, somatic complaints, and rule-breaking behaviors (Parola et al., 2020), have also been detected in the dreams of adolescents and young adults (Parrello et al., 2021; Sommantico et al., 2021).

Considering the abovementioned literature findings, and according to Villani et al. (2020), we investigated the situational future time perspective of Italian university students during the pandemic, considered a collective traumatic event, assessable through a perceived change in future orientation. Some dispositional traits reported in the literature as associated with both future orientation and psychological well-being were also considered: (a) optimism, which refers to positive expectations about the future (Scheier and Carver, 1993), influences immediate acts (Zhang et al., 2007), and helps to overcome expected obstacles that arise in the pursuit of the goal (Zhang and Fishbach, 2010); (b) the belief that life has meaning which is considered relevant in the transition to adulthood (e.g., Steger et al., 2006; Shterjovska and Achkovska-Leshkovska, 2014; Baikeli et al., 2021; Lasota and Mróz, 2021; Zambelli et al., 2022; Osorio Guzmán et al., 2022a) and is usually associated with well-being (McMahan and Renken, 2011), life satisfaction (Joshanloo, 2018), and happiness (Li et al., 2019); finally, (c) dispositional aggression was found useful to also consider (Sommantico et al., 2015), hypothesizing that the pandemic may have amplified aggressive traits, by some of its characteristics. Finally, since most of the studies on the psychological well-being of students during the pandemic reported the presence of internalizing symptoms, it was considered to survey levels of anxiety, depression, and stress. The present study, therefore, had the following objectives:

- to develop a self-report questionnaire measuring the situational future time perspective specifically taking into account whether and how young Italian young adults, particularly university students, perceived and recognized the impact of the pandemic on their plans for the future: the Pandemic, Time, and Future Scale (PTFS);
- to test whether and to what extent such a dramatic experience, assessed by constructing specific “pandemic indicators” (see Measures section), interacted with certain dispositional traits such as optimism, belief that life has meaning, aggressive tendencies, and future time perspective in the aforementioned target group;
- to test whether and to what extent the situational variables (“pandemic indicators” and perceived future perspective at the time) and dispositional variables (optimism, sense of life, aggression, and usual future perspective) affected the target group’s psychological well-being, in terms of anxiety, stress, and depression.

## 2 Materials and methods

### 2.1 Procedure and participants

Participants were recruited in Italy via the Internet, through advertisements on social media, between April 1, 2021, and May 31, 2021—that was the most dramatic phase of the pandemic in Italy, by the number of hospitalizations and deaths—according to the following two criteria: being between 18 and 35 years old and compliant with the quarantine/isolation measures. All data were collected through self-report questionnaires, using an Internet-based survey (Hewson et al., 2016). To improve this web-based sampling, we also used snowball sampling. To do this, we first asked recruited participants to identify other potential respondents from their social network; these potential participants were also asked to nominate individuals from their social network, and so on. Participation in the study was voluntary, anonymous, and unpaid. All participants included in the study gave consent to participate on the first page of the survey. The informed consent included detailed information about the aims and procedures of the study, its confidentiality, and the anonymity of the responses. Participants completed, in the following order: (a) a socio-demographic questionnaire, the Life Orientation Test-Revised (LOT-R; Scheier and Carver, 1985); (b) the Meaning in Life Questionnaire (MLQ; Steger et al., 2006); (c) the Aggression Questionnaire (AQ; Buss and Perry, 1992); (d) the Future Time Perspective Scale for Adolescents and Young Adults (FTPS-AYA; Lyu and Huang, 2016); (e) the Pandemic, Time, and Future Scale (PTFS); and (f) the Depression Anxiety Stress Scale - 21 (DASS-21; Lovibond and Lovibond, 1995).

There were 389 respondents (73.5% women; ages 18–35 years, *M* = 23.5, *SD* = 4.4). Most participants (83.8%) lived in a “red zone,” subject to the highest levels of restrictive measures. Regarding the educational level, 54.2% of participants had completed secondary school, and 45.7% had completed a university degree. The majority of participants (78.1%) were university students, and 31.4% were employed. Participants who modified their work habits because of the restrictive measures were 41.6, and 4.9% were laid off because of the pandemic. Most participants (83.8%) lived with their families both before (77.9%) and during the pandemic (84.8%). For most participants (91%), the pandemic affected plans for the future, and 43.7% reported that their families had suffered economic damage due to the pandemic. Participants who had been affected by COVID-19 were 9.8%, participants who knew someone infected by COVID-19 were 94.9%, and participants whose relatives or friends died of COVID-19 were 25.7%. Finally, participants who were quarantined because they were living with someone ill with COVID-19 were 21.3%.

### 2.2 The development procedure of the questionnaire

Pandemic, Time and Future Scale (PTFS) was created to fill the gap in previous time perspective instruments for measuring situational time perspective. In the first phase, the initial set of items was created. Two steps were followed: (a) item generation through the assessment of indicators of one established domain of situational time perspective; (b) content validity assessed through the evaluation of three expert judges (clinical researchers). An agreement of at least 80% between judges was considered adequate to retain each item. This process resulted in a set of 7 items. In the second phase, items were administered to 70 young adults to assess whether those items were understandable for the target population. In addition, the content of the items was further discussed in 4 focus groups of 15 young adult participants each. No changes have been made at this stage.

### 2.3 Measures

*Socio-demographic questionnaire*. Questions on both socio-demographic variables and specific “pandemic indicators” were included in the socio-demographic questionnaire. Respondents provided socio-demographic data about age, gender, educational level, and profession. To operationalize the pandemic experience into situational variables, specific “pandemic indicators” were identified and constructed: region of residence at high pandemic risk (“red zones” vs. “orange zones,” or “yellow zones”), modifications of work habits related to the COVID-19 pandemic, living conditions (e.g., with parents or friends), economic harms (e.g., own or family members’ layoffs), changes in plans for the future, the experience of COVID-19 illness and quarantine (own or family member’s), knowledge of people affected by or died from COVID-19. Participants were then also requested to report information about these indicators.

The *Pandemic, Time, and Future Scale* (PTFS) was the instrument *ad hoc* constructed to measure situational future time perspective. At the time of administration, the scale included the 7 items created that assessed whether the experience of the pandemic has hurt the organization of time and vision of the future. Participants are asked to respond according to a 5-point Likert-type scale (ranging from 1 = “*Completely disagree*” to 5 = “*Completely agree*”). Examples of items are: “This pandemic has negatively changed me” and “Compared to before the pandemic, I feel I have wasted time in achieving my goals.”

The *Future Time Perspective Scale for Adolescents and Young Adults* (FTPS-AYA; Lyu and Huang, 2016) was chosen to measure dispositional future time perspective. It is a 28-item self-report instrument that assesses future time perspective, understood as a personality trait involving people’s thoughts, feelings, and actions related to their future and is structured on six subscales: (a) Future Negative (7 items referred to the future viewed with fear, anxiety, and hopelessness); (b) Future Positive (5 items referred to the future viewed with hope for success and optimism); (c) Future Confusion (4 items referred to the future that appears uncertain and unclear); (d) Future Perseverant (5 items referred to the future that can be achieved by working hard to overcome failure and adversity); (e) Future Perspicuity (3 items referred to a clear vision of the future); and (f) Future Planning (4 items referred to goal setting and commitment to future rewards). Participants are asked to respond according to a 5-point Likert-type scale (ranging from 1 = “*Strongly disagree*” to 5 = “*Strongly agree*”). Examples of items are: “I believe I am able to control my future through my own efforts” and “I move forward every day without making plans.” Given that in previous studies on the Italian population (Konidari and Benetton, 2019; Konidari, 2021) good internal consistency has been found only for the Future Positive and the Future Negative subscales, only these two subscales were used in the current study, reporting a Cronbach’s *α, respectively,* of 0.92 and 0.87.

The *Life Orientation Test-Revised* [LOT-R; Scheier and Carver, 1985; Italian adaptation and validation by Giannini et al. (2008)] is a 10-item self-report instrument assessing dispositional optimism. Participants are asked to respond according to a 5-point Likert-type scale (ranging from 0 = “*Strongly disagree*” to 5 = “*Strongly agree*”). Examples of items are: “I hardly believe that things are going in my favor” and “I am always optimistic about my future.” The authors of the Italian version of the LOT-R reported good internal consistency (Giannini et al., 2008). In the present study, Cronbach’s *α* was 0.78.

The *Meaning in Life Questionnaire* [MLQ; Steger et al., 2006; Italian adaptation and validation by Di Fabio (2014)] is a 10-item self-report instrument assessing dispositional meaning in life on two subscales: (a) Presence (5 items), and (b) Search (5 items). Participants are asked to respond according to a 7-point Likert-type scale (ranging from 1 = “*Absolutely true*” to 7 = “*Absolutely untrue*”). Examples of items are: “I am aware of what makes my life meaningful” and “I am always looking for something to make my life meaningful.” The author of the Italian version of the MLQ reported good internal consistency (Di Fabio, 2014). In the present study, Cronbach’s *α* was 0.86 for MLQ Presence and 0.88 for MLQ Search.

The *Aggression Questionnaire* (AQ; Buss and Perry, 1992; Italian adaptation and validation, Sommantico et al., 2008), is a 29-item self-report instrument assessing dispositional aggression on four subscales: (a) Physical Aggression (9 items); (b) Verbal Aggression (5 items); (c) Anger (7 items); and (d) Hostility (8 items). Participants are asked to respond according to a 5-point Likert-type scale (ranging from 1 = “*Extremely uncharacteristic of me*” to 5 = “*Extremely characteristic of me*”). Examples of items are: “I often feel like a barrel of gunpowder ready to explode” and “I do not hesitate to resort to violence to defend my rights.” The authors of the Italian version of the AQ reported good internal consistency and age invariance of the factor structure (Sommantico et al., 2015). In the present study, Cronbach’s *α* ranged from 0.71 to 0.77 for each subscale and was 0.87 for the total score.

The *Depression Anxiety Stress Scale - 21* [DASS-21; Lovibond and Lovibond, 1995; Italian adaptation and validation by Bottesi et al. (2015)], was chosen to assess self-perceived psychological well-being/discomfort. It is a 21-item self-report instrument assessing depression, anxiety, and stress on three subscales: (a) Depression (7 items); (b) Anxiety (7 items); and (c) Stress (7 items). Participants are asked to rate the frequency and severity of depression, anxiety, and stress symptoms on a 4-point Likert-type scale (ranging from 0 = “*Did not apply to me at all*” to 3 = “*Applied to me very much, or most of the time*”) in the past week. Examples of items are: “(In the last 7 days) I had difficulty relaxing,” “There was nothing to give me enthusiasm,” and “I felt I was worth little as a person.” The authors of the Italian version of the DASS-21 reported good internal consistency (Bottesi et al., 2015). In the present study, Cronbach’s *α* was 0.91 for Depression, 0.87 for Anxiety, 0.88 for Stress, and 0.94 for the total score.

### 2.4 Data analyses plan

First, the database was cleaned, as indicated by Streiner et al. (2015). Then, survey data were entered into the SPSS 28.0 (IBM Corp, 2021) and Mplus8 (Muthén and Muthén, 2017) databases and checked and verified by project staff for accuracy.

A one factor solution with 7 items was tested through Confirmatory Factor Analysis (CFA). The maximum likelihood estimator was used. To assess the adequacy of model to the data, the following fit indices were calculated: chi-squared distribution and the degrees of freedom (*χ^2^*/*df*; in a range from 2 to 5), Comparative Fit Index (CFI; > 0.90), Tucker and Lewis Index (TLI; > 0.90), Root Mean Square Error of Approximation (RMSEA; considered good if the values are <0.05, reasonable if they are <0.08, and average if they are <0.10), and Standardized Root Mean Square Residual (SRMR; < 0.09; Tucker and Lewis, 1973; Bentler, 1990; Hu and Bentler, 1995; McDonald and Ho, 2002; Kline, 2005). The reliability analyses were computed using Cronbach’s *α* and considered to be satisfactory if the values were > 0.70 (Nunnally and Berstein, 1995).

To assess the relationship between study variables, correlations analyses were conducted using Pearson’s coefficient (*r*; between 0.10 and 0.29 = small association; between 0.30 and 0.49 = medium association; and > 0.50 = large association; *p* < 0.05). Group differences were verified through ANOVA and Tukey tests (*p* < 0.05). Effect sizes were measured through Eta-square (*η^2^*; small ≥0.01; medium ≥0.059; large ≥0.138; Cohen, 1988). Multiple regression analyses were conducted using standardized *β* coefficients and *R^2^* coefficients (*p* < 0.05).

## 3 Results

### 3.1 Descriptive statistics

Means, Standard Deviations, and Cronbach’s α between study variables are shown in Table 1. The mean for LOT-R was 17.2 (*SD* = 5.2); the means for MLQ Presence and MLQ Search were, respectively, 18.0 (*SD* = 6.6) and 25.8 (*SD* = 6.9); the means for AQ ranged between 1.8 for Physical Aggression (*SD* = 9.7) and 3.2 for Verbal Aggression (*SD* = 0.8); the mean for FTPS Positive and FTPS Negative were, respectively, 3.1 (*SD* = 0.9) and 3.0 (*SD* = 1.0); the mean for PTFS was 2.7 (*SD* = 0.9); and the means for Depression, Anxiety, and Stress were, respectively, 20.9 (*SD* = 11.8), 16.3 (*SD* = 11.3), and 26.8 (*SD* = 9.8).

**Table 1:** Means, standard deviations, and Cronbach’s *α*.

	Females (*N* = 286)		Males (*N* = 103)		Total sample (*N* = 389)		
	*M* (range)	*SD*	*M* (range)	*SD*	*M* (range)	*SD*	*α*
LOT-R	16.8 (6–29)	5.3	18.3 (6–30)	4.8	17.2 (6–30)	5.2	0.78
MLQ Presence	18.3 (3–34)	6.7	17.4 (1–34)	6.4	18.0 (1–34)	6.6	0.86
MLQ Search	26.4 (6–35)	6.7	24.1 (5–35)	7.2	25.8 (5–35)	6.9	0.88
Physical Aggression	1.7 (1–4.3)	0.7	2.1 (1–4.1)	0.8	1.8 (1–4.3)	0.7	0.71
Verbal Aggression	3.2 (1.2–5)	0.9	3.3 (1.6–5)	0.7	3.2 (1.2–5)	0.8	0.71
Rage	2.7 (1.1–4.9)	0.8	2.6 (1–4.6)	0.8	2.7 (1–4.9)	0.8	0.73
Hostility	2.9 (1.1–5)	0.9	2.9 (1–4.9)	0.8	2.9 (1–5)	0.9	0.77
AQ Total Score	2.7 (1.3–4.4)	0.6	2.7 (1.4–4)	0.6	2.7 (1.3–4.4)	0.7	0.87
FTPS Positive	3.0 (1–5)	0.9	3.2 (1–5)	0.9	3.1 (1–5)	0.9	0.92
FTPS Negative	3.1 (1–5)	1.0	2.9 (1–5)	1.1	3.0 (1–5)	1.0	0.87
PTFS	2.6 (1–5)	0.9	2.9 (1–5)	1.0	2.7 (1–5)	0.9	0.87
Depression	21.5 (0–42)	11.8	19.1 (0–42)	11.7	20.9 (0–42)	11.8	0.91
Anxiety	17.4 (0–42)	11.6	13.2 (0–42)	9.9	16.3 (0–42)	11.3	0.87
Stress	28.1 (0–42)	9.5	23.2 (0–42)	9.8	26.8 (0–42)	9.8	0.88

### 3.2 Confirmatory factor analysis, reliability, construct validity, and convergent validity of the pandemic, time, and future scale (PTFS)

CFA was utilized to verify the appropriateness of the proposed one-factor model, using the maximum likelihood estimation method. The model was tested on a sample of 389 subjects with no missing data, resulting in high goodness of fit scores (*χ^2^/df* = 2.56; *RMSEA* = 0.024 [0.022–0.027]; *CFI* = 0.94; *TLI = 0*.96; *SRMR* = 0.083). These findings support the hypothesis of a one-factor structure of the PTFS.

Cronbach’s *α* was 0.85, thus indicating satisfactory reliability (See Table 2).

**Table 2:** Pandemic, time, and future scale—PTFS items, factor loadings, and communalities (*N* = 389).

Item	Factor loading	*R^2^*	Skewness/Kurtosis
1. This pandemic experience has changed me in a negative way	0.624	0.070	−0.166/−0.941
2. Compared to before the pandemic, I feel that I have wasted time in achieving my goals	0.711	0.110	−0.600/−0.952
3. Compared to before the pandemic, I feel a greater sense of uncertainty about the future	0.673	0.069	0.793/−0.615
4. Compared to before the pandemic, I feel that I have less hope that I will be able to finish, on time, paths previously taken	0.714	0.087	0.352/−1.145
5. In light of the current situation, I have more concerns about my future	0.653	0.056	0.868/−0.373
6. I believe that the future will offer me good opportunities	0.544	0.071	−0.158/−0.572
7. I have confidence in the future	0.613	0.086	−0.170/−0.788
Cronbach’s *α* = 0.85			

Items 6 and 7 were reversed to obtain a single score measuring the negative impact of the pandemic on time management and future time vision.

PTFS construct validity and convergent validity were supported as showed by the correlational analyses between PTFS and FTPS scales reported in the next section.

### 3.3 Correlations and group differences

Zero-order correlations between participants’ age and the measures are presented in Table 3. Results indicate that: LOT-R was significantly negatively correlated with Depression (*r* = −0.60; *p* < 0.01), Anxiety (*r* = −0.38; *p* < 0.01), and Stress (*r* = −0.45; *p* < 0.01); MLQ Presence was significantly negatively correlated with Depression (*r* = −0.41; *p* < 0.01), Anxiety (*r* = −0.11; *p* < 0.05) and Stress (*r* = −0.18; *p* < 0.01); MLQ Search was significantly positively correlated with Depression (*r* = 0.39; *p* < 0.01), Anxiety (*r* = 0.26; *p* < 0.01), and Stress (*r* = 0.37; *p* < 0.01); AQ total score was significantly positively correlated with Depression (*r* = 0.41; *p* < 0.01), Anxiety (*r* = 0.43; *p* < 0.01), and Stress (*r* = 0.52; *p* < 0.01); FTPS Positive was significantly negatively correlated with Depression (*r* = −0.51; *p* < 0.01), Anxiety (*r* = −0.17; *p* < 0.05), and Stress (*r* = −0.29; *p* < 0.01); FTPS Negative was significantly positively correlated with Depression (*r* = 0.66; *p* < 0.01), Anxiety (*r* = 0.31; *p* < 0.01), and Stress (*r* = 0.47; *p* < 0.01); and PTFS was significantly negatively correlated with Depression (*r* = −0.64; *p* < 0.01), Anxiety (*r* = −0.31; *p* < 0.05), and Stress (*r* = −0.47; *p* < 0.01). Zero-order correlations between the PTFS and the FTPS indicated that PTFS was significantly positively correlated with FTPS Positive (*r* = 0.69; *p* < 0.01) and significantly negatively correlated with FTPS Negative (*r* = −0.78; *p* < 0.01).

**Table 3:** Zero-order correlations between participants’ age and the measures (*N* = 389).

	1	2	3	4	5	6	7	8	9	10	11	12	13	14	15
1. Age	–														
2. LOT-R	0.15**	–													
3. MLQ Presence	0.07	0.51**	–												
4. MLQ Search	−0.18**	−0.31**	−0.02	–											
5. Physical Aggression	−0.04	−0.12*	−0.08	0.08	–										
6. Verbal aggression	−0.10*	−0.09	0.03	0.09	0.38**	–									
7. Anger	−0.19**	−0.28**	−0.07	0.26**	0.51**	0.64**	–								
8. Hostility	−0.29**	−0.55**	−0.31**	0.32**	0.28**	0.31**	0.54**	–							
9. AQ Total Score	−0.21**	−0.34**	−0.14**	0.25**	0.69**	0.77**	0.88**	0.70**	–						
10. FTPS Positive	−0.03	0.62**	0.63**	−0.21**	−0.01	0.06	−0.07	−0.27**	−0.10	–					
11. FTPS Negative	−0.07	−0.60**	−0.46**	0.38**	0.13*	0.08	0.22**	0.44**	0.28**	−0.75**	–				
12. PTFS	0.12*	0.59**	0.50**	−0.31**	−0.11*	−0.09	−0.20**	−0.43**	−0.28**	0.69**	−0.78**	–			
13. Depression	−0.22**	−0.60**	−0.41**	0.39**	0.19**	0.18**	0.33**	0.56**	0.41**	−0.51**	0.66**	−0.64**	–		
14. Anxiety	−0.21**	−0.38**	−0.11*	0.26**	0.20**	0.22**	0.40**	0.47**	0.43**	−0.17**	0.34**	−0.31**	0.62**	–	
15. Stress	−0.23**	−0.45**	−0.18**	0.37**	0.18**	0.29**	0.51**	0.59**	0.52**	−0.29**	0.48**	−0.47**	0.74**	0.70**	–

*p = 0.05; **p = 0.01.

ANOVA showed significant gender differences. Indeed, male participants reported significantly higher scores than female participants on LOT-R (*M_F_* = 16.8 vs. *M_M_* = 18.3; *F*_(1, 388)_ = 6.672, *p* = 0.01; *η^2^* = 0.02), the AQ Physical Aggression subscale (*M_F_* = 1.7 vs. *M_M_* = 2.1; *F*_(1, 388)_ = 15.371, *p* < 0.01; *η^2^* = 0.04), and PTFS (*M_F_* = 2.6 vs. *M_M_* = 2.9; *F*_(1, 388)_ = 5.432, *p* < 0.05; *η^2^* = 0.01). On the contrary, female participants reported higher scores than males on the MLQ Research subscale (*M_F_* = 26.4 vs. *M_M_* = 24.1; *F*_(1, 388)_ = 8.719, *p* < 0.01; *η^2^* = 0.02), Anxiety (*M_F_* = 17.4 vs. *M_M_* = 13.2; *F*_(1, 388)_ = 10.705, *p* < 0.01; *η^2^* = 0.03), and Stress (*M_F_* = 28.1 vs. *M_M_* = 23.2; *F*_(1, 388)_ = 19.689, *p* < 0.01; *η^2^* = 0.05).

ANOVA also showed significant differences concerning career. Indeed, student participants reported significantly lower scores than working participants on the AQ Physical Aggression subscale (*M_I_* = 1.8 vs. *M_II_* = 2.1; *F*_(1, 349)_ = 11.530, *p* < 0.01; *η^2^* = 0.03), as well as significantly higher scores than working participants for Depression (*M_I_* = 21.5 vs. *M_II_* = 18.5; *F*_(1, 388)_ = 4.256, *p* < 0.05; *η^2^* = 0.01) and Stress (*M_I_* = 27.6 vs. *M_II_* = 23.9; *F*_(1, 388)_ = 10.095, *p* < 0.05; *η^2^* = 0.02).

ANOVA and Tukey tests also showed significant differences concerning the region of residence. Indeed, participants living in “red zones” reported significantly higher scores than participants living in “yellow zones” or “orange zones” for Depression (*M_I_* = 19.3, *M_II_* = 15.8, and *M_III_* = 21.6; *F*_(1, 349)_ = 4.582, *p* < 0.05; *η^2^* = 0.02) and Stress (*M_I_* = 25.6, *M_II_* = 23.2, and *M_III_* = 27.3; *F*_(1, 349)_ = 3.296, *p* < 0.05; *η^2^* = 0.02).

Furthermore, ANOVA showed significant differences in terms of cohabitation conditions. Indeed, participants living with parents during the pandemic reported significantly higher scores than participants living alone, with a partner, or with friends for Rage (Before *M_I_* = 2.7, *M_II_* = 2.5, *M_III_* = 2.8, and *M_IV_* = 2.4; *F*_(1, 349)_ = 3.487, *p* < 0.05; *η^2^* = 0.03; During *M_I_* = 2.3, *M_II_* = 2.6, *M_III_* = 2.8, and *M_IV_* = 2.3; *F*_(1, 349)_ = 2.929, *p* < 0.05; *η^2^* = 0.02), Hostility (Before *M_I_* = 3.0, *M_II_* = 2.5, *M_III_* = 3.2, and *M_IV_* = 2.5; *F*_(1, 349)_ = 7.347, *p* < 0.01; *η^2^* = 0.05; During *M_I_* = 2.5, *M_II_* = 2.7, *M_III_* = 3.0, and *M_IV_* = 2.4; *F*_(1, 349)_ = 5.147, *p* < 0.01; *η^2^* = 0.04), and Depression (Before *M_I_* = 16.2, *M_II_* = 19.3, *M_III_* = 21.7, and *M_IV_* = 17.7; *F*_(1, 349)_ = 2.643, *p* < 0.05; *η^2^* = 0.02; During *M_I_* = 10.9, *M_II_* = 17.5, *M_III_* = 21.5, and *M_IV_* = 20.7; *F*_(1, 349)_ = 4.439, *p* < 0.01; *η^2^* = 0.03).

Moreover, ANOVA showed significant differences regarding living conditions. Indeed, participants who have a non-shared room at their disposal reported significantly lower scores on the MLQ Research subscale than participants who do not have a private room (*M_I_* = 25.1 vs. *M_II_* = 27.6; *F*_(1, 388)_ = 9.722, *p* < 0.01; *η^2^* = 0.02), while those with a private room also reported significantly higher scores on FTPS Negative subscale (*M_I_* = 3.0 vs. *M_II_* = 3.2; *F*_(1, 388)_ = 4.718, *p* < 0.05; *η^2^* = 0.01) and PTFS (*M_I_* = 2.8 vs. *M_II_* = 2.5; *F*_(1, 388)_ = 6.915, *p* < 0.01; *η^2^* = 0.02).

ANOVA also showed significant differences regarding economic damages related to the pandemic. Indeed, participants reporting that their family suffered economic damages related to the pandemic also reported lower scores than participants whose plans were not affected by the pandemic on LOT-R (*M_I_* = 16.6 vs. *M_II_* = 17.6; *F*_(1, 388)_ = 4.276, *p* < 0.05; *η^2^* = 0.01), in addition to significantly higher scores on MLQ Research (*M_I_* = 16.6 vs. *M_II_* = 17.6; *F*_(1, 388)_ = 6.926, *p* < 0.01; *η^2^* = 0.02), Rage (*M_I_* = 2.9 vs. *M_II_* = 2.6; *F*_(1, 388)_ = 9.487, *p* < 0.01; *η^2^* = 0.02), Hostility (*M_I_* = 3.1 vs. *M_II_* = 2.8; *F*_(1, 388)_ = 15.251, *p* < 0.01; *η^2^* = 0.04), FTPS Negative (*M_I_* = 3.2 vs. *M_II_* = 2.9; *F*_(1, 388)_ = 7.840, *p* < 0.01; *η^2^* = 0.02), PTFS (*M_I_* = 3.2 vs. *M_II_* = 2.9; *F*_(1, 388)_ = 15.994, *p* < 0.01; *η^2^* = 0.04), Depression (*M_I_* = 23.2 vs. *M_II_* = 19.1; *F*_(1, 388)_ = 11.854, *p* < 0.01; *η^2^* = 0.03), Anxiety (*M_I_* = 19.3 vs. *M_II_* = 13.9; *F*_(1, 388)_ = 23.135, *p* < 0.01; *η^2^* = 0.06), and Stress (*M_I_* = 29.8 vs. *M_II_* = 24.5; *F*_(1, 388)_ = 36.063, *p* < 0.01; *η^2^* = 0.07).

Moreover, ANOVA showed significant differences regarding the influence of the pandemic on plans for the future. Indeed, participants who reported that the quarantine affected their plans for the future also reported significantly lower scores than participants whose plans were not affected by the pandemic on LOT-R (*M_I_* = 17.0 vs. *M_II_* = 18.9; *F*_(1, 388)_ = 4.489, *p* < 0.05; *η^2^* = 0.01), in addition to significantly higher scores on MLQ Research (*M_I_* = 26.0 vs. *M_II_* = 23.3; *F*_(1, 388)_ = 5.054, *p* < 0.05; *η^2^* = 0.01), Rage (*M_I,_* = 2.7 vs. *M_II_* = 2.4; *F*_(1, 388)_ = 4.627, *p* < 0.05; *η^2^* = 0.01), Hostility (*M_I_* = 3.0 vs. *M_II_* = 2.6; *F*_(1, 388)_ = 7.338, *p* < 0.01; *η^2^* = 0.02), PTFS (*M_I_* = 3.0 vs. *M_II_* = 2.9; *F*_(1, 388)_ = 16.334, *p* < 0.01; *η^2^* = 0.04), Depression (*M_I_* = 21.5 vs. *M_II_* = 14.5; *F*_(1, 388)_ = 11.389, *p* < 0.01; *η^2^* = 0.03), and Stress (*M_I_* = 27.6 vs. *M_II_* = 18.6; *F*_(1, 388)_ = 29.263, *p* < 0.01; *η^2^* = 0.07).

Finally, ANOVA showed significant differences regarding being quarantined. Indeed, participants being quarantined reported significantly higher scores than participants not being quarantined on Anxiety (*M_I_* = 18.8 vs. *M_II_* = 15.6; *F*_(1, 388)_ = 5.360, *p* < 0.05; *η^2^* = 0.01) and Stress (*M_I_* = 29.3 vs. *M_II_* = 26.1; *F*_(1, 388)_ = 7.284, *p* < 0.01; *η^2^* = 0.02).

### 3.4 Regression analyses

Based on previous results, several hierarchical multiple regression analyses were conducted to determine the extent to which each variable contributed to the models predicting Depression, Anxiety, and Stress (see Tables 4–6).

**Table 4:** Hierarchical multiple regression analysis summary predicting depression (*N =* 389).

Step and predictor variable	*β*	*R^2^*	*ΔR^2^*
1. Age, gender	0.076	0.054	0.054*
2. LOTR	−0.582	0.381	0.326*
3. MLQ Research	0.216	0.422	0.041*
4. AQ	0.190	0.452	0.030*
5. FTPS Negative	0.423	0.557	0.105*
6. PTFS	−0.198	0.571	0.014*

*p < 0.001.

**Table 5:** Hierarchical multiple regression analysis summary predicting anxiety (*N =* 389).

Step and predictor variable	*β*	*R^2^*	*ΔR^2^*
1. Age, gender	0.152	0.067	0.067***
2. LOTR	−0.343	0.181	0.174***
3. MLQ Research	0.125	0.194	0.186**
4. AQ	0.317	0.278	0.269***
5. FTPS Negative	0.127	0.288	0.277*
6. PTFS	0.022	0.288	0.275

*p < 0.05; **p < 0.01; ***p < 0.001.

**Table 6:** Hierarchical multiple regression analysis summary predicting stress (*N =* 389).

Step and predictor variable	*β*	*R^2^*	*ΔR^2^*
1. Age, gender	0.206	0.095	0.095**
2. LOTR	−0.410	0.257	0.252**
3. MLQ Research	0.223	0.301	0.294**
4. AQ	0.384	0.424	0.416**
5. FTPS Negative	0.235	0.456	0.448**
6. PTFS	−0.150	0.465	0.455*

*p < 0.01; **p < 0.001.

After controlling for differences in age and gender, the addition of LOTR to the prediction of Depression led to a statistically significant increase in *R^2^* of 0.326, *F*(_1, 385_) = 202.981, *p* < 0.001. The addition of MLQ Research to the prediction of Depression led to a statistically significant increase in *R^2^* of 0.041, *F*(_1, 384_) = 27.001, *p* < 0.001. The addition of AQ to the prediction of Depression led to a statistically significant increase in *R^2^* of 0.030, *F*(_1, 383_) = 21.135, *p* < 0.001. The addition of FTPS Negative to the prediction of Depression led to a statistically significant increase in *R^2^* of 0.105, *F*(_1, 382_) = 90.708, *p* < 0.001. Finally, the addition of PTFS to the prediction of Depression led to a statistically significant increase in *R^2^* of 0.014, *F*(_1, 381_) = 12.822, *p* < 0.001. The full model of age, gender, LOT-R, MLQ Research, AQ, FTPS Negative, and PTFS for predicting depression was statistically significant, *R*^2^ = 0.571, *F*(_7, 388_) = 72.559, *p* < 0.001, adjusted *R*^2^ = 0.564.

After controlling for differences in age and gender, the addition of LOT-R to the prediction of Anxiety led to a statistically significant increase in *R^2^* of 0.114, *F*(_1, 385_) = 55.408, *p* < 0.001. The addition of MLQ Research to the prediction of Anxiety led to a statistically significant increase in *R^2^* of 0.014, *F*(_1, 384_) = 6.543, *p* < 0.01. The addition of AQ to the prediction of Anxiety led to a statistically significant increase in *R^2^* of 0.084, *F*(_1, 383_) = 44.564, *p* < 0.001. The addition of FTPS Negative to the prediction of Anxiety led to a statistically significant increase in *R^2^* of 0.009, *F*(_1, 382_) = 5.074, *p* < 0.05. Finally, the addition of PTFS to the prediction of Anxiety did not lead to a statistically significant increase in *R^2^*, *F*(_1, 381_) = 0.095, *p* = 0.758. Thus, excluding PTFS, the model of age, gender, LOT-R, MLQ Research, AQ, and FTPS Negative to predict Anxiety was statistically significant, *R*^2^ = 0.288, *F*(_6, 388_) = 25.732, *p* < 0.001, adjusted *R*^2^ = 0.277.

After controlling for differences in age and gender, the addition of LOTR to the prediction of Stress led to a statistically significant increase in *R^2^* of 0.162, *F*(_1, 385_) = 84.167, *p* < 0.001. The addition of MLQ Research to the prediction of Stress led to a statistically significant increase in *R^2^* of 0.043, *F*(_1, 384_) = 23.852, *p* < 0.001. The addition of AQ to the prediction of Stress led to a statistically significant increase in *R^2^* of 0.123, *F*(_1, 383_) = 81.784, *p* < 0.001. The addition of FTPS Negative to the prediction of Stress led to a statistically significant increase in *R^2^* of 0.033, *F*(_1, 382_) = 22.895, *p* < 0.001. Finally, the addition of PTFS to the prediction of Stress led to a statistically significant increase in *R^2^* of 0.008, *F*(_1, 381_) = 5.870, *p* < 0.01. The full model of age, gender, LOTR, MLQ Research, AQ, FTPS Negative, and PTFS to predict Stress was statistically significant, *R*^2^ = 0.465, *F*(_7, 388_) = 47.257, *p* < 0.001, adjusted *R*^2^ = 0.455.

## 4 Discussion

As emerged from the literature review on the topic, the psychological well-being of young adults was particularly challenged during the pandemic, especially in terms of increased levels of anxiety, depression, post-traumatic symptoms, compulsive behaviors, and social isolation (e.g., Glowacz and Schmits, 2020; Parola et al., 2020; Varma et al., 2021; Galvin et al., 2022; Giotsa, 2022; Hawes et al., 2022; Ludwig-Walz et al., 2022; Foster et al., 2023). This vulnerability is likely related to the fact that the pandemic has negatively affected some developmental tasks of this age, including building personal and professional futures. In particular, the COVID-19 pandemic appears to have significantly affected the future time perspective of university students, because its sudden and unexpected spread forced governments to take infection containment measures that drastically affected their routines and schedules. Consequently, there has been an amplification of experiences of uncertainty, anxiety, and worry concerning the future (e.g., Nowakowska, 2020; Carey et al., 2023; Fynes-Clinton and Addis, 2023). Moreover, it is important to point out that in Italy the future time perspective of young people has long been studied (Leccardi, 2005; Crocetti et al., 2012) also because of some contextual specificities. Indeed, young Italian adults postpone more than others taking on commitments typical of adult life and have greater difficulty in planning (Sica et al., 2016).

Questioning whether these substantial modifications introduced by the COVID-19 pandemic produced significant changes in previous trends, the present study investigated the relationships between situational aspects related to the pandemic experience (such as “pandemic indicators” and situational future time perspective) and dispositional traits (such as optimism, belief that life makes sense, appropriate aggressive tendencies, and dispositional future time perspective), considered as possible protective factors for psychological well-being (depression, anxiety, and stress) in a sample of Italian young adults during the second wave of the COVID-19 pandemic.

Confirmatory factor analysis of PTFS, indicating the perceived negative impact of the pandemic on one’s view of the future, confirmed the hypothesized one-factor structure of the instrument, as indicated by the good levels of the model-data fit indexes. Furthermore, Cronbach’s alpha value (0.85), indicated satisfactory reliability. The results also supported the instrument’s construct validity. Indeed, the results show a significant positive correlation between a high pandemic-related situational future time perspective and dispositional positive future time perspective score and a significant negative correlation between a high pandemic-related situational future time perspective and dispositional negative future time perspective score. This could indicate that it was precisely the young adults with a dispositional positive perspective toward the future who perceived the pandemic-induced negative change in their orientation toward the future more, making a painful reality check. The Uruguayan study by Loose et al. (2022) did not find the same perhaps because the research took place in a less dramatic phase of the pandemic, when the number of infected and dead was not worrisome. Instead, our study took place at a still very dramatic phase for Italy (April–May 2021). After the end of the epidemic control, the young adults with a dispositional positive perspective toward the future have not recovered from the negative changes caused by the epidemic.

The results of descriptive analyses, correlations, and group differences show that the “positive” dispositional traits—such as optimism, belief that life has meaning, and dispositional positive future time perspective—significantly negatively correlated with internalizing disorders—such as depression, anxiety, and stress. While being still in search of meaning in life, a highly general aggressive tendency, and dispositional negative future time perspective significantly positively correlated with depression, anxiety, and stress.

Furthermore, the perception of the strong negative impact of the pandemic on one’s plans for the future, in terms of pandemic-related situational negative future time perspective, significantly negatively correlated with depression, anxiety, and stress. It is possible to hypothesize that those who recognize, without denying the negative impact of the ongoing situation, can take the necessary measures to cope with the emergency and protect their mental health. This would be in line with studies showing that even a negative future time perspective or one oriented to the “here and now” can play a protective function, prompting one to adopt preventive healthy behaviors, provided they are associated with the perception of one’s vulnerability and high risk (Zancu et al., 2022) and specific dispositional traits (Mioni et al., 2022), including the belief that the present is rich in value and that current behaviors are determinative for the future (e.g., Sobol et al., 2020; Zambelli et al., 2022).

The ANOVA results provide interesting evidence related not only to gender and profession but also to the “pandemic indicators.” Indeed, male participants reported significantly higher scores in pandemic-related situational negative future perspective but also in two dispositional traits, namely optimism and tendency to physical aggression. Female participants reported higher scores than males in being still searching for meaning in life, as well as in anxiety and stress levels. These results are in line with the literature findings showing greater psychological distress of women during the pandemic (e.g., Ranta et al., 2020; Servidio et al., 2022; Fulcher et al., 2023; Zhu et al., 2023). As for profession, college students reported significantly lower scores than working peers in the tendency for physical aggression and significantly higher scores in depression and stress. Thus, they seem to exhibit greater overall malaise, not easy to explain. Indeed, some studies have pointed to the influence of technostress related to distance learning (Galvin et al., 2022), others to concern related to delays in academic activities (Cao et al., 2020), and still others to the interaction with additional risk factors, such as female gender, minority membership, or economic disadvantage (Browning et al., 2021).

As for the “pandemic indicators,” they all allow for some interesting reflections. Indeed, participants living in “red zones” (higher risk of infection and high restrictions) reported significantly higher scores than participants living in “yellow zones” or “orange zones” (lower risk of infection and low restrictions) in levels of depression and stress. Regarding living conditions, those who spent the lockdown with their parents reported significantly higher scores than participants who lived alone, with partners, or friends, in the levels of anger, hostility, and depression. In contrast with Skinner et al. (2022), reporting that adolescents living with their parents during the lockdown felt more “protected,” in our study, young adults forced to spend more time at home with their parents, giving up some of their autonomy, experienced negative feelings. Furthermore, participants reporting that their family suffered economic harm related to the pandemic also reported lower scores in optimism than participants who were not affected economically by the pandemic, as well as significantly higher scores in life sense seeking, hostility, dispositional negative future time perspective, pandemic-related situational negative future time perspective, depression, anxiety, and stress. Thus, this economic situational variable seems to be particularly “powerful,” as highlighted by other studies (e.g., Ranta et al., 2020; Ganson et al., 2021), especially when associated with negative dispositional traits such as low optimism and still searching for meaning in life. One can imagine that it weighs more heavily for those young people who belong to disadvantaged socioeconomic strata, who enjoyed fewer job protections during the pandemic. This is in line with literature findings pointing out how unevenly the pandemic has produced distress by amplifying pre-existing inequalities (Carey et al., 2023).

Concerning changes in personal plans for the future, participants who responded affirmatively reported significantly lower scores than participants whose plans were not affected by the pandemic in optimism and significantly higher scores in still searching for meaning in life, anger, hostility, and pandemic-related situational negative future time perspective, as well as higher levels of depression and stress. Finally, according to previous studies (Cao et al., 2020), participants who quarantined because they were living with someone sick with COVID-19 reported significantly higher scores than non-quarantined participants in anxiety and stress levels. It is possible to hypothesize that the need to organize themselves at home, without the support of the health care institution, along with concern for their own and their loved one’s health and survival, became additional risk factors. In conclusion, having verified that the “pandemic indicators” were found to be significant for the well-being of the participating young adults, allows for a deeper analysis of the pandemic event, and makes it possible to argue that it was much more than a serious health event and fell unevenly on the population, without governments being able to take it into account.

Hierarchical multiple regression analyses, performed to determine the extent to which each variable contributed to the prediction models for depression, anxiety, and stress, showed that the full model of age, sex, LOT-R, MLQ Research, AQ, negative FTPS, and PTFS was statistically significant for predicting depression and stress, while for predicting anxiety the same model was statistically significant, but with the exclusion of PTFS. Thus, the pandemic-related situational future time perspective would seem to be irrelevant in predicting anxiety. It is possible to hypothesize that this anxiety is nonetheless widespread in young adults, especially in the form of social anxiety (Jefferies and Ungar, 2020), and that being aware of the special situation of the moment does not affect it so significantly, unlike dispositional traits that color the future black.

Moreover, the prevalence of university students in the sample may be relevant in this sense. Indeed, different studies have demonstrated that this population is more likely to experience anxiety (e.g., Asif et al., 2020; Tan et al., 2023).

A strength of this study is the attempt to research the interaction of the material and psychological effects of the pandemic on young adults during a difficult period: namely, the second wave of transmission, focusing on future time perspective. Furthermore, to our knowledge, the PTFS is the only existing measure investigating situational future time perspective during the lockdown period in the young adult population. Therefore, it may be a useful instrument for investigating this specific field of interest in post-COVID research as well.

However, the present study has its limitations. The first general limitation is related to sampling. Convenience sampling implies specific biases, such as volunteers’ bias, related to the special characteristics of individuals who voluntarily participate in a study. Another possible bias in the study is that of the mono-method, related to the fact that having assessed all variables of the study by using self-report instruments, there may be inflation in observed associations. Furthermore, our sample was not balanced for gender. A further limitation is the fact that, out of a total sample of 389 subjects, only 87 are between the ages of 26 and 35, thus making it impossible to make comparisons between age groups that could have allowed for analyzing and understanding of potential differences about the impact of the pandemic on subjects of different ages. Finally, the same sample was used for confirmatory factor analyses useful for psychometric validation of the instrument and subsequent analyses. Taken together, these limitations do not allow for the generalizability of the results to the entire population of Italian young adults. Future research perspectives include comparing the Italian data with those collected in other countries, partly already published (Osorio Guzmán et al., 2022a,b, 2024), and the planning of longitudinal research design, which could allow for causal inferences, not possible in this study given its cross-sectional nature.

In the past 3 years, since the outbreak of the COVID-19 pandemic, numerous studies have attempted to sketch a picture of the psychological effects this complex event has had especially on populations considered particularly vulnerable. This study adds useful insights because it shows the interplay between dispositional and situational factors in a specific group such as young adult students, in a particular context such as Italy, and in an important area such as future time perspective. The results seem to indicate that the pandemic, as a health event, but also as a socio-economic-political event, has had a significant impact on the development of new generations, the outcomes of which will be more precisely assessable over time. The “emergency” situation certainly did not help in constructing “differentiated” pandemic management according to the diverse needs of the population, but it is to be hoped that this experience will help focus on the specific needs of all ages and conditions. In particular, even from the results of the present study, it is clear that young adults, and especially university students, need educational and economic policies that help them develop confidence in the future and in their ability to build it. Furthermore, our findings show that the pandemic has differently affected people with varying personality features, highlighting the necessity of programs tailored to different types of young adults. Therefore, the results of this study could be useful for policymakers to better understand mental health challenges in time of crises, such as the COVID-19 pandemic, as well as to implement support and preventive interventions in workplaces and educational institutions. For instance, introducing personalized psychological counseling could be useful to reinforce the future orientation of young adults addressing personal needs and qualities of university students, workers and unemployed. In this vein, by tailoring interventions based on specific personality traits, counselors can empower young adults’ capacity to cope with emergency making their personal characteristics an asset in resisting stressful situations and in preserving confidence in the future. These initiatives may improve the mental health of young adults and foster their capacity to believe in a positive future, thus strengthening their psychological well-being and resilience in time of crises.

## Data availability statement

The original contributions presented in the study are included in the article/supplementary material, further inquiries can be directed to the corresponding author.

## Ethics statement

The study complied with the ethical standards of the American Psychological Association in the treatment of human research participants and was in accordance with the provisions of the 1995 Declaration of Helsinki and subsequent modifications. Written informed consent was obtained from the individual(s) for the publication of any potentially identifiable data included in this article. The study was reviewed and approved by the Psychological Research Ethics Committee of the Department of Humanities of the University of Naples Federico II (prot. no. 12/2021).

## Author contributions

SP: Conceptualization, Formal analysis, Investigation, Supervision, Writing – original draft, Writing – review & editing. JP: Conceptualization, Investigation, Methodology, Writing – original draft, Writing – review & editing. LS: Conceptualization, Investigation, Supervision, Writing – review & editing. BR: Conceptualization, Investigation, Supervision, Writing – review & editing. AP: Investigation, Methodology, Supervision, Writing – review & editing. GR: Investigation, Methodology, Writing – original draft. EF: Investigation, Methodology, Writing – original draft. MS: Conceptualization, Formal analysis, Investigation, Methodology, Supervision, Writing – review & editing.
